# Music-induced Mood Biases Decision Strategies during the Ultimatum Game

**DOI:** 10.3389/fpsyg.2016.00453

**Published:** 2016-03-30

**Authors:** Hwanjun Chung, Eun Jung Lee, You Jin Jung, Sang Hee Kim

**Affiliations:** Department of Brain and Cognitive Engineering, Korea UniversitySeoul, South Korea

**Keywords:** mood, decision-making, ultimatum game, fairness, happiness

## Abstract

Recently, an increasing attempt has been made to understand the influence of mood on socioeconomic decision-making. We tested in this study whether an unpleasant mood would lead to unfavorable decisions more frequently than a pleasant mood, and whether decisions under different moods can be explained in different ways. Healthy volunteers were assigned to either a pleasant or unpleasant mood group and listened to musical excerpts to induce pleasant or unpleasant mood. Both groups completed the ultimatum game as a responder with an unacquainted partner who was actually a confederate. The proposer’s offers were made in six different ratios of split (1:9, 2:8, 3:7, 4:6, 5:5, 6:4) in a preprogramed manner unbeknownst to the participants. After the completion of the task as a responder, the participant rated subjectively perceived fairness and emotional feelings about each split of offer. The statistical results showed that the unpleasant mood group rejected unfair offers more often compared to the pleasant mood group. Self-reported ratings of perceived fairness and emotional feelings did not statistically differ between the two groups. Interestingly, however, only in the unpleasant mood group, rejection rates of unfair offers were negatively correlated with perceived fairness. Both the pleasant and unpleasant mood groups showed a negative correlation between rejection rates of unfair offers and self-reported happiness. These results suggest a possibility that different decision strategies operate under different mood during a socioeconomic exchange.

## Introduction

Economic decisions are often made in a social context where two or more people interact with each other. Individuals’ socioeconomic decision-making has been studied extensively with the ultimatum game (Güth et al., 1982; Bolton and Zwick, 1995; Suleiman, 1996; Güth and Van Damme, 1998; Murnighan and Saxon, 1998). In the ultimatum game, two players are engaged: a proposer and a responder. The proposer has to decide how an amount of money should be split to make an offer to the responder. The responder must either accept or reject the offer. If the responder accepts the offer, both players receive the money as proposed. On the other hand, if the responder rejects the offer, neither player receives any money. Based on the standard economic models, the proposer offers as little as possible and the responder accepts any offers greater than zero (Selten, 1975). Surprisingly, however, the empirical findings show that the proposer often offers an equal split of the total amount and the responder typically rejects offers 30% below the total amount (Güth et al., 1982). This economically irrational decision suggests that people not only care about reward gain but also about reciprocity and fairness (Camerer et al., 2005; Gintis, 2005; Fehr and Camerer, 2007). Violations of such fairness norms tend to elicit negative emotional reactions, such as insult and anger, and these negative emotions motivate responders to reject the offers (Pillutla and Murnighan, 1996).

Previous studies have shown that responders’ rejection rate can be modulated by various factors including the origin of the money (house money vs. earned money; Dannenberg et al., 2012), level of testosterone (Burnham, 2007), and gender of the players (Solnick and Schweitzer, 1999; Eckel and Grossman, 2001). Moreover, a handful of studies have shown that induced mood also influences decisions to accept or reject during the ultimatum game (Harlé and Sanfey, 2007; Andrade and Ariely, 2009; Forgas and Tan, 2013). Specifically, when an unpleasant mood was induced by a negative film, responders rejected unfair offers more frequently compared to when a pleasant mood was induced (Forgas and Tan, 2013). Responders induced with sadness and disgust also showed increased rejection of unfair offers compared to those induced with amusement (Harlé and Sanfey, 2007). The consensus that emerged in previous studies of mood effect was that unpleasant mood increases rejection of unfair offers compared to pleasant mood. This mood-dependent change in rejection rate was explained by information processing bias under different moods (Forgas and Tan, 2013). According to Bless and Fiedler’s (2006) assimilative-accommodative processing model, negative mood signals problems in the environment and, thus, motivates individuals to pay more attention to externally driven information. In contrast, positive affect signals that the environment is benign and orients people to pay more attention to internal experiences (Bless and Fiedler, 2006). Therefore, the responders under negative mood were thought to be more sensitive to external information, such as a violation of the reciprocal fairness norm, while those under positive mood were thought to be more sensitive to internal motivation when making decisions to accept or reject unfair offers (Forgas and Tan, 2013). Although these explanations are reasonable to account for the difference in rejection between pleasant and unpleasant mood, there has been little attempt to investigate individual differences within groups to determine further whether fairness and emotional reactions reliably explain within-group variances.

The current study was designed to investigate how mood influences socioeconomic decision-making using the ultimatum game. Unlike previous studies that typically used video films or still images to induce mood (Harlé and Sanfey, 2007; Moretti and Di Pellegrino, 2010; Forgas and Tan, 2013), in this study we used musical excerpts. As compared with mood induced by films or images, music-induced mood was reported to have the greatest personal relevance (Ellard et al., 2012) and, therefore, was thought to have greater impact on other cognitive processes. We proposed the following hypotheses focusing on unfair offers because fair offers were found to generate unitary accept responses: (1) individuals induced with unpleasant mood would reject unfair offers more often compared with those induced with pleasant mood, (2) individuals with unpleasant mood would show greater tendency to reject as they perceive offers to be less fair, and (3) individuals with pleasant mood would reject offers more often as they are more emotionally upset about the offers. We recruited healthy volunteers and assigned them to either the pleasant or unpleasant mood group. Participants in the pleasant group listened to musical excerpts prepared to induce positive mood and participants in the unpleasant mood group listened to musical excerpts prepared to induce negative mood. Both groups completed an ultimatum game as a responder with an unacquainted partner, who was actually a confederate who played the role of a proposer. Participants were asked to decide to reject or accept offers provided by the proposer. Offers were made in six different ratios of split. Participants also rated felt fairness and emotional feelings about each split of offer at the end of the task.

## Materials and Methods

### Participants

A total of 40 healthy volunteers (22 females, 21.55 ± 1.84 years; 18 males, 21.55 ± 3.89 years) were recruited by posting an advertisement on a community website. All volunteers were college students and reported no past or current diagnosis of neurological and psychiatric disorders. They were randomly assigned to either the pleasant or unpleasant music conditions. This study was conducted in accordance with the Declaration of Helsinki and the procedures were approved by the local institutional review board. All participants provided informed consent and were monetarily compensated for their time. Two participants from the pleasant group and one from the unpleasant group were excluded from data analyses because they responded with accept (*n* = 1) or reject (*n* = 2) decisions for all offers during the ultimatum game.

### Mood Induction by Musical Excerpts

To induce pleasant and unpleasant moods, we used musical excerpts identified from a previous study (Roy et al., 2008). Three 1-min pleasant excerpts were from “Love and Happiness” by Ernst Ranglin, “William Tell Overture” by Rossini, and “French Cancan” by Canissimo. Three 1-min unpleasant excerpts were from “Pendulum Music” by Sonic Youth, “The Threshold of Deafening Silence” by Paul Dolden, and “Fascicles” by the Thirteen Ghosts with Derek Bailey and Thurston Moore. To equate loudness range across musical excerpts, all selected excerpts were sound-normalized. Participants assigned to the pleasant group listened to the three pleasant excerpts and those assigned to the unpleasant group listened to the three unpleasant ones.

### Ultimatum Game

The ultimatum game was programmed using E-prime 2.0 (Psychology Software Tools Inc., Pittsburgh, PA, USA). Participants were instructed to play an economic decision-making game trying to earn as much money as possible. They were told that there were two players: the proposer and the responder. The proposer distributes 10,000 KRW (∼9 USD) between him/herself and the responder. The responder decides to either accept or reject the distribution. If accepted, the proposer and responder both receive exactly the amount as distributed by the proposer. If rejected, both participants earn zero. Participants were told that the role would be randomly assigned by the computer, and the assigned role would not change throughout the game. In reality, participants always played the responder and a confederate played the proposer. Participants were told that they would receive the sum of the money they earned in eight randomly chosen trials, in addition to their participation compensation. In fact, all received the same amount of extra money (∼4.5 USD). Each trial in the task started with a picture of a written text stating “Your partner is making an offer,” lasting an average of 5 s. Then, a picture of an offer was presented and remained until a response was made. Participants pressed “1” for acceptance and “2” for rejection on a keyboard. Finally, participants were presented with a feedback screen for 4 s showing the outcome. A total of 32 offers were made including fair and unfair splits. There were four offers of 6 (responder):4 (proposer), four offers of 5:5, four offers of 4:6, eight offers of 3:7, eight offers of 2:8, and four offers of 1:9. The different offers were presented in pseudo-random order.

### Procedure

Participants were tested individually and the whole procedure took about 1 h. Upon arrival, participants were greeted by an experimenter, and they provided informed consent after it was explained that they would participate in two separate studies: one examining the relationship between personality and musical preference and the other studying socioeconomic decision-making. They were told that they would complete the music preference study first and another participant, who would arrive soon, would join the subsequent economic decision-making study. Then, they answered several questions in written form about their musical preferences, such as favorite music type, favorite artist, and time spent listening to music. These questions were prepared to mask the real purpose of the music treatment. The participants’ mood was assessed with the Positive and Negative Affect Schedule (PANAS; Watson et al., 1988). Participants in the pleasant group listened to the three 1-min pleasant musical excerpts and those in the unpleasant group listened to the three 1-min unpleasant musical excerpts. At the end of each excerpt, participants rated subjective preference for the music on a 5-point Likert scale (1, not at all prefer; 5, strongly prefer). After listening to all music excerpts, the participants’ mood states were again evaluated using the PANAS.

**FIGURE 1:**
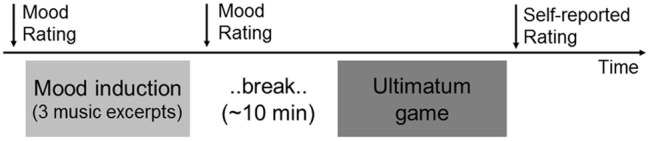
**Time course of mood induction and ultimatum game task**.

At the end of the music preference task, participants were told that the partner for the second study would be arriving in 5 min and they received instructions about the ultimatum game task. The task partner was actually a same-sex confederate who arrived in exactly 5 min. The confederate sat at a separate desk, which was visually blocked from the participant. The confederate partner signed a consent form and received the same instructions about the task. Although visually blocked, the participants could hear interactions between the confederate partner and experimenter. The task started to run and participants were informed by the computer screen that they were assigned to the responder role. Participants were always assigned to the responder role, although they were ostensibly told that the assignment was random. After the completion of the ultimatum game task, participants completed self-reported ratings of fairness and emotional reactions (happiness, envy, anger, contempt; 1, very weak; 7, very strong) on each split of offers (**Figure 1**). Participants were then debriefed and thanked for their time. During debriefing, participants were asked to guess the purpose of the two studies. None of them guessed correctly about the relationship between music listening and the ultimatum game. All participants believed that they played the ultimatum game with a real partner.

## Results

### Manipulation Check: Mood Induction

We confirmed that music preference scores differed across the groups, *t*(35) = 8.10, *p* < 0.0001. As expected, the pleasant group (*M* = 4.29, *SD* = 0.85) preferred the music more than the unpleasant group (*M* = 2.31, *SD* = 1.07).

Analyses of variance (ANOVA) were conducted separately on positive and negative affect scores with time (pre- vs. post-induction) as the within-subject factor and group as the between-subject factor. Positive and negative affect scores were separately calculated as the sum of the five pleasant descriptors (interested, enthusiastic, active, inspired, excited) and the sum of the five unpleasant descriptors (distressed, upset, irritable, afraid, hostile), respectively.

**FIGURE 2:**
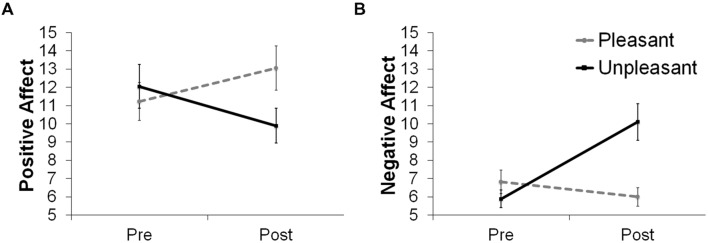
**(A)** Positive and **(B)** negative affect changes after listening to musical excerpts.

A significant Time × Group interaction was found for negative affect scores, *F*(1,35) = 19.61, *p* < 0.001. Follow-up *t*-tests indicated that the unpleasant group reported increased negative affect after mood induction relative to pre-induction, *t*(18) = 4.10, *p* = 0.001, whereas the pleasant group reported marginally decreased negative affect after mood induction relative to pre-induction, *t*(17) = 1.87, *p* = 0.08. A significant Time × Group interaction was also found for positive affect scores, *F*(1,35) *=* 6.77, *p* = 0.013. Follow-up *t*-tests indicated that the pleasant group reported increased positive affect after mood induction relative to pre-induction, *t*(17) = 2.22, *p* = 0.04, whereas the unpleasant group reported an insignificant decrease in positive affect, *t*(18) = 1.69, *p* = 0.11 (**Figure 2**). Exploratory analyses confirmed that preference ratings and mood change scores were not associated with rejection rates of any of the offers (*r*s < 0.3).

### Rejection Rate and Self-Reported Measures across Groups

#### Rejection Rates

**FIGURE 3:**
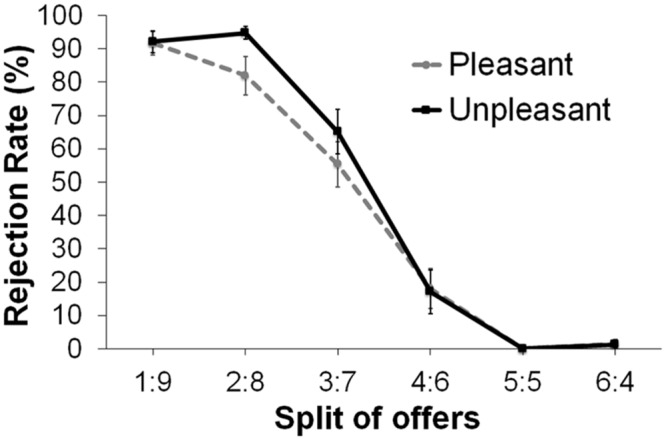
**Rejection rates as a function of offer split across the two groups**.

Rejection rates of each offer across the groups are presented in **Figure 3**. A 6 (offer) × 2 (group) × 2 (sex) mixed ANOVA was conducted. A significant main effect of offer was found, *F*(5,175) = 234.65, *p* < 0.001. As offers were less favorable, participants rejected more (1:9 = 2:8 > 3:7 > 4:6 > 5:5 = 6:4; for statistical results, all *t*s > 3, *p*s < 0.001). No other main effect or interaction effects were found (*F*s < 1.5).

**FIGURE 4:**
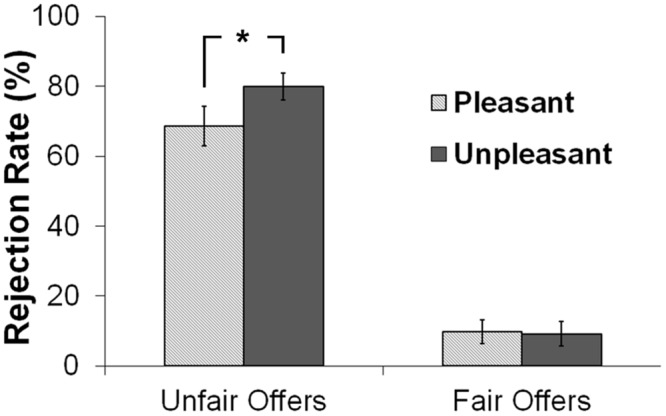
**Rejection rates across the two groups as a function of fairness.** Error bars indicate the standard error of the mean. The asterisk indicates statistical significance (*p* < 0.05).

To further our specific hypothesis that rejection of unfair offers would be modulated by music-induced mood, we first grouped splits into two offer conditions: fair and unfair conditions. For the unfair condition, we grouped the 2:8 and 3:7 offers, and for the fair condition, we grouped the 4:6 and 6:4 offers. Because 73% of participants rejected the 1:9 offers all of the time and 100% of participants accepted the 5:5 offers all of the time, these two offers were excluded in this grouping. A 2 (offer: fair, unfair) × 2 (group) × 2 (sex) mixed ANOVA revealed a main effect of offer, *F*(1,33) = 344.45, *p* < 0.0001. Unfair offers (*M* = 74.44, *SD* = 20.97) were rejected more often than fair offers (*M* = 9.45, *SD* = 14.55). A significant Offer × Group interaction was found, *F*(1,33) = 4.26, *p* = 0.047. Follow-up *t*-tests revealed that the unpleasant group rejected unfair offers more often than the pleasant group, *t*(35) = 1.68 *p* = 0.05, one-tail (**Figure 4**). The group difference was not significant for fair offers (*t* < 1). No other main or interaction effects were found (*Fs* < 1.5).

#### Self-Reported Measures

**FIGURE 5:**
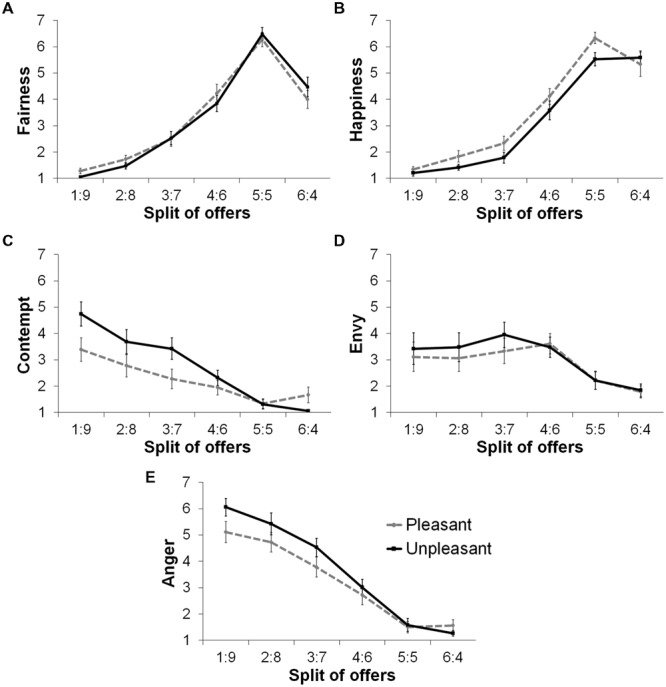
**Self-reported fairness and felt emotional reactions to each split of offer by group.** Fairness **(A)**, Happiness **(B)**, Contempt **(C)**, Envy **(D)**, and Anger **(E)**.

We tested whether perceived fairness and emotional reactions differed across fair and unfair offers (**Figure 5**). A 2 (offer: fair, unfair) × 2 (group) mixed ANOVA was conducted for each self-report measure. Significant differences between fair and unfair conditions were found for all measures. Participants felt less fairness, *F*(1,35) = 133.08, *p* < 0.0001, less happy, *F*(1,35) = 172.32, *p* < 0.0001, more envious, *F*(1,35) = 7.32, *p* < 0.05, more contempt, *F*(1,35) = 22.22, *p* < 0.0001, and more anger, *F*(1,35) = 111.31, *p* < 0.0001, for unfair offers compared with fair offers. For contempt, there was a significant Offer × Group interaction, *F*(1,35) = 4.358, *p* = 0.044, revealing that the pleasant group felt less contempt than the unpleasant group for unfair offers, *t*(35) = 1.789, *p* = 0.041, one-tail, but not for fair offers (*t* < 1). No main effects of group or interaction effects were found for other measures (*F*s < 1.5).

### Correlation between Rejection Rates and Self-Reported Measures

To examine whether a rejection decision was associated with degrees of fairness and emotional reactions experienced for unfair offers, we conducted within-group correlation analyses between rejection rates and self-reported ratings separately for each group. We limited this analysis to the unfair offers because fair offers did not show group differences in rejection (**Table 1**).

**Table 1:** Correlation coefficients between self-reported ratings and rejection rates.

		Self-reported ratings				
	Group	Fairness	Happiness	Envy	Contempt	Anger
Rejection Rate of unfair offers	Pleasant	-0.427	-0.603^∗^	-0.325	-0.046	0.184
	Unpleasant	-0.473^∗^	-0.627^∗^	-0.035	0.209	0.191

*The asterisk indicates statistical significance (*p* < 0.05).*

Rejection rates of unfair offers were negatively correlated with felt fairness (*r* = -0.473, *p* = 0.041) and felt happiness (*r* = -0.627, *p* = 0.004) in the unpleasant group, indicating that the unpleasant group rejected more often as they perceived the offer to be less fair and were less happy. The pleasant group also showed a negative correlation between felt happiness and rejection rates (*r* = -0.603, *p* = 0.008) indicating that they rejected more often as they perceived the offer less happily. No additional significant correlations were found. Given that perceived fairness was associated with rejection of unfair offers only in the unpleasant group, we performed a moderation analysis to determine whether the link between fairness and rejection was moderated by mood, using the Hayes Process tool (Hayes, 2013). We failed to find a significant interaction between fairness and group (*F* < 1).

## Discussion

In the current study, we investigated differences in rejection rates of unfair offers during a socioeconomic decision-making game between individuals with pleasant and unpleasant mood. We found that the unpleasant mood group rejected unfair offers more often relative to the pleasant mood group. Furthermore, the responders’ rejection rates of unfair offers were negatively correlated with self-reported happiness for both the pleasant and unpleasant groups. Additionally, in the unpleasant group, rejection rates of unfair offers were negatively correlated with perceived fairness. These results are partially consistent with our predictions and suggest that different decision strategies may operate under different mood during a socioeconomic exchange.

Consistent with previous results (Harlé and Sanfey, 2007; Andrade and Ariely, 2009; Srivastava et al., 2009; Moretti and Di Pellegrino, 2010; Forgas and Tan, 2013), we found differences in rejection rates between the pleasant and unpleasant groups. The pleasant group accepted unfair offers more often than the unpleasant group. We also found that the unpleasant group felt greater contempt than the pleasant group. However, unlike previous research showing that negative emotion, such as anger and contempt, was a critical factor for the decision to reject the offer (Pillutla and Murnighan, 1996; Nowak et al., 2000; Sanfey et al., 2003; Van’t Wout et al., 2006), we found no associations between rejection rates and contempt in both mood groups. Instead, we found that the unpleasant group, but not the pleasant group, rejected the unfair offers more often as they perceived the offer to be less fair. This result is partially consistent with the processing effects model of mood suggesting that negative mood directs attention to external information whereas positive mood directs attention to internal information (Bless and Fiedler, 2006). Reciprocal fairness is a universally accepted social norm and people normally expect to be treated fairly (Camerer et al., 2005; Gintis, 2005; Fehr and Camerer, 2007). Under unpleasant mood, our participants may have become sensitive to external information, such as the violation of the fairness norm. Thus, if participants considered that the offered amount violated the fairness norm, they might have tended to react unfavorably to the proposer. The fact that both groups reported similar levels of perceived fairness and yet this association between fairness and rejection was found only in the unpleasant group suggests a possibility that mood may systematically bias information processing by directing attention to a particular piece of information among many, which, in turn, influences the operation of decision strategies during socioeconomic exchange. However, this view should be taken with caution. Notably, our moderation analysis that examined whether the relationship between perceived fairness and rejection rates was moderated by mood failed to find a significant interaction between perceived fairness and rejection rates. The small sample size may account for the lack of significance. Alternatively, the effect of induced mood could have been attenuated by explicit awareness of mood; there is evidence that explicit awareness of mood reduces its influence on subsequent judgments and performance (Schwarz, 2004). Therefore, a future study with a bigger sample and a more cautious procedure is warranted to determine further the moderation effect of mood on the link between fairness and socioeconomic decision-making.

Unlike other studies investigating mood, we used musical excerpts to induce pleasant and unpleasant mood. Music, as compared with other mood-induction tools, such as still images and video clips, is considered to generate affect that is perceived as being more personally relevant, and that is associated with more elaborate psychological processes, such as episodic memory (Baumgartner et al., 2006; Ellard et al., 2012). Therefore, there could be qualitative differences in the effect of mood induced by music and that induced by visual media, which could influence other psychological and cognitive processes in different ways. It would be worthwhile to investigate in a controlled comparison study how mood induced by different media types differentially interacts with the processes underlying socioeconomic decision-making.

## Conclusion

The current study showed that negative mood leads to more frequent rejection of unfair offers during a socioeconomic exchange compared to positive mood. We speculate that responder’s decisions are influenced by the proposer’s violation of the fairness norm under unpleasant mood. This finding suggests a possibility that mood leads to a selective adoption of different decision strategies during a socioeconomic decision game. Further studies, however, are required to clearly define the mechanism by which different moods exert their role in socioeconomic exchange in different ways.

## Author Contributions

SK conceived and designed the study, analyzed and interpreted the data, wrote and revised the manuscript, and approved the final version for publication. HC conceived and designed the study, collected, analyzed and interpreted the data, wrote the first draft, and approved the final version for publication. EL collected and analyzed the data, wrote the first draft, and approved the final version for publication. YJ designed the study, interpreted the data, wrote the first draft, and approved the final version for publication. SK, HC, EL, and YJ agreed to be accountable for all aspects of the work in ensuring that questions related to the accuracy or integrity of any part of the work are appropriately investigated and resolved.

## Conflict of Interest Statement

The authors declare that the research was conducted in the absence of any commercial or financial relationships that could be construed as a potential conflict of interest.
